# A Critical Assessment of Vector Control for Dengue Prevention

**DOI:** 10.1371/journal.pntd.0003655

**Published:** 2015-05-07

**Authors:** Nicole L. Achee, Fred Gould, T. Alex Perkins, Robert C. Reiner, Amy C. Morrison, Scott A. Ritchie, Duane J. Gubler, Remy Teyssou, Thomas W. Scott

**Affiliations:** 1 Department of Biological Sciences and Eck Institute for Global Health, University of Notre Dame, Notre Dame, Indiana, United States of America; 2 Department of Entomology, North Carolina State University, Raleigh, North Carolina, United States of America; 3 Fogarty International Center, National Institutes of Health, Bethesda, Maryland, United States of America; 4 Department of Epidemiology and Biostatistics, Indiana University School of Public Health, Bloomington, Indiana, United States of America; 5 Department of Entomology and Nematology, University of California, Davis, Davis, California, United States of America; 6 United States Naval Medical Research Unit, No. 6, Iquitos, Peru; 7 College of Public Health, Medical and Veterinary Sciences, James Cook University, Cairns, Australia; 8 Emerging Infectious Diseases Program, Duke-NUS Graduate Medical School, Singapore, Singapore; 9 Partnership for Dengue Control, Fondation Mérieux, Lyon, France; Pediatric Dengue Vaccine Initiative, UNITED STATES

## Abstract

Recently, the Vaccines to Vaccinate (v2V) initiative was reconfigured into the Partnership for Dengue Control (PDC), a multi-sponsored and independent initiative. This redirection is consistent with the growing consensus among the dengue-prevention community that no single intervention will be sufficient to control dengue disease. The PDC's expectation is that when an effective dengue virus (DENV) vaccine is commercially available, the public health community will continue to rely on vector control because the two strategies complement and enhance one another. Although the concept of integrated intervention for dengue prevention is gaining increasingly broader acceptance, to date, no consensus has been reached regarding the details of how and what combination of approaches can be most effectively implemented to manage disease. To fill that gap, the PDC proposed a three step process: (1) a critical assessment of current vector control tools and those under development, (2) outlining a research agenda for determining, in a definitive way, what existing tools work best, and (3) determining how to combine the best vector control options, which have systematically been defined in this process, with DENV vaccines. To address the first step, the PDC convened a meeting of international experts during November 2013 in Washington, DC, to critically assess existing vector control interventions and tools under development. This report summarizes those deliberations.

## Introduction

Dengue virus (DENV) causes more human morbidity and mortality than any other arboviral disease. Recent estimates are that approximately 390 million people are infected each year and 96 million manifest with clinically apparent disease [1]. The World Health Organization (WHO) called for an increased focus on dengue prevention in its 2013 report on neglected tropical diseases. Dengue’s explosive epidemic potential and the worldwide increase in dengue cases was noted. Specifically, dengue has experienced a 30-fold increase in incidence over the past 50 years that shows no sign of slowing down. The report calls for evaluation and integration of current intervention strategies to achieve a 50% reduction in dengue mortality and 25% reduction in dengue morbidity by 2020 [2].

DENV is transmitted from one person to another by *Aedes* mosquitoes, principally *Aedes aegypti* [3]. Human infections can result in a spectrum of diseases ranging from mild, self-limiting febrile illness to classic dengue fever to shock syndrome and death [4], but it has been recognized that the majority of infections are mild or show no clinical manifestations. Presently, there are no commercially available antivirals or dengue vaccines, though progress on their development is encouraging [5–11]. Dengue prevention, therefore, is currently limited to mosquito control. A few well-documented successes indicate that rigorously applied vector control can reduce DENV transmission and disease. During the 1950s and 1960s a regional program across much of the Caribbean and Central and South America dramatically reduced *Ae*. *aegypti* populations, resulting in striking reductions in human yellow fever and DENV infections and purported elimination of *Ae*. *aegypti* from most of the region by the early 1970s [12,13]. Vector control programs for dengue control in Singapore during the 1970s and 1980s [14] and in Cuba during the 1980s and 1990s [15–17] are similarly recognized as public health successes, but they, too, were unfortunately not sustainable. The inability of these countries to sustain their gains is considered, at least in part, to be the consequence of diminished herd immunity [18]. This is worth emphasizing, particularly in the context of envisaged vaccination programmes, which may help overcome the problem of vector control sustainability by elevating herd immunity [19]. Less dramatic results, for example during 2000–2010 in Peru, were seen by the successful use of emergency vector control to reduce the force of DENV infection [20], i.e., per capita risk of human infection [19]. There is, however, considerable frustration because vector control has failed to prevent epidemics and DENV’s expanding geographic distribution [21]. Although the concept of vector control is reasonable, control must be early in an outbreak or strategically applied during inter-epidemic periods to prevent escalation in transmission and successful broad scale application has been difficult to achieve (Box 1).

**Box 1. Key Learning Points from Vector Control for Dengue Prevention**

Success in reducing the public health burden of dengue will require a multi-pronged approach that includes developing the underlying theory of effective dengue control, continuing to review and assess existing interventions and strategies, and gathering new empirical data that tests fundamental concepts and strategies. This is best achieved through:

- Controlled experimental studies are urgently needed to assess the health impact of dengue vector control based on epidemiological and entomological metrics. Interventions under development are required to address this issue. The gap is greatest for existing tools and strategies, which were developed in an era when epidemiologic assessments were not carried out.
- Although greater emphasis should be placed on proactive strategies that aim to prevent, diminish, or eliminate transmission, dengue outbreaks occur and response requires attention to development of tools and strategies that can be rapidly deployed and have swift impact.
- Dengue is a growing problem in modern mega-urban centers that span across countries and regions. Scaling up from successful small-scale experimental trials to broad-scale public health application is among the biggest challenges for improving mosquito control for dengue prevention.
- There is a growing consensus that eliminating dengue as a public health burden can only be achieved by integrating vector control with vaccines. Prioritizing the vector interventions based on their potential to prevent disease will facilitate determining how to combine the best options with DENV vaccines.

### Mission of the Partnership for Dengue Control

The initiative Vaccines to Vaccination (v2V) was established in 2009 to facilitate delivery of DENV vaccines following regulatory approval. After publication of results from a phase 2b clinical dengue vaccine trial in Thailand indicating varying levels of vaccine efficacy [9], the program was reconfigured into the Partnership for Dengue Control (PDC), a multi-sponsored and independent initiative. Redirection of the program was consistent with the growing consensus among the dengue prevention community that no single intervention will be sufficient to control dengue disease. This is due to heterogeneities in mosquito vector, viral pathogen, and human host factors that drive the complexity of transmission. It was decided, therefore, that, for the greatest likelihood of sustained dengue prevention, the PDC mission should be to promote development and implementation of multiple innovative, integrated, and synergistic interventions.

The PDC’s expectation is that when an effective DENV vaccine is commercially available, the public health community will continue to rely on vector control because the two strategies complement and enhance one another [19]. A dengue vaccine can be used to artificially elevate herd immunity and vector control to lower the force of infection. Theory and results from field studies with other vector-borne pathogens support the power of simultaneously targeting the vector and pathogen. The global malaria burden has been reduced using anti-*Plasmodium* drugs in conjunction with insecticide-treated bed nets [22–25]. Lymphatic filariasis (LF) is more rapidly and efficiently managed when anti-parasite drugs are combined with vector control than when drugs alone are used [26–32]. As has been the case for malaria and lymphatic filariasis, guidance for how to best integrate interventions against a complicated, multi-strain pathogen with complex transmission dynamics, like DENV, will benefit from preliminary, theoretical assessment with mathematical and simulation models [33,34].

The use of multiple strategies for vector-borne disease prevention is the basis of WHO’s Integrated Vector Management (IVM) program for control of mosquito vectors, including those that transmit DENV [35]. An element of IVM is decision-making guided by operational research and entomological and epidemiological surveillance and evaluation. The expectation is that as field data becomes available for dengue vector interventions, so, too, will the ability to refine guiding principles for implementation and maximizing impact. This evidence will serve as the basis for assessment, challenging traditional paradigms and changes in the standards for delivery of vector control and/or vaccines.

As a first step, the PDC initiated a study to assess state-of-the-art vector control for dengue. Goals were to critically review different tools and strategies, identify those approaches with the greatest potential for positive public health impact, identify key missing information required for guiding successful vector management, and propose productive ways for filling knowledge gaps and advancing dengue prevention. This report summarizes those deliberations.

### Assessment Process

**Fig 1:**
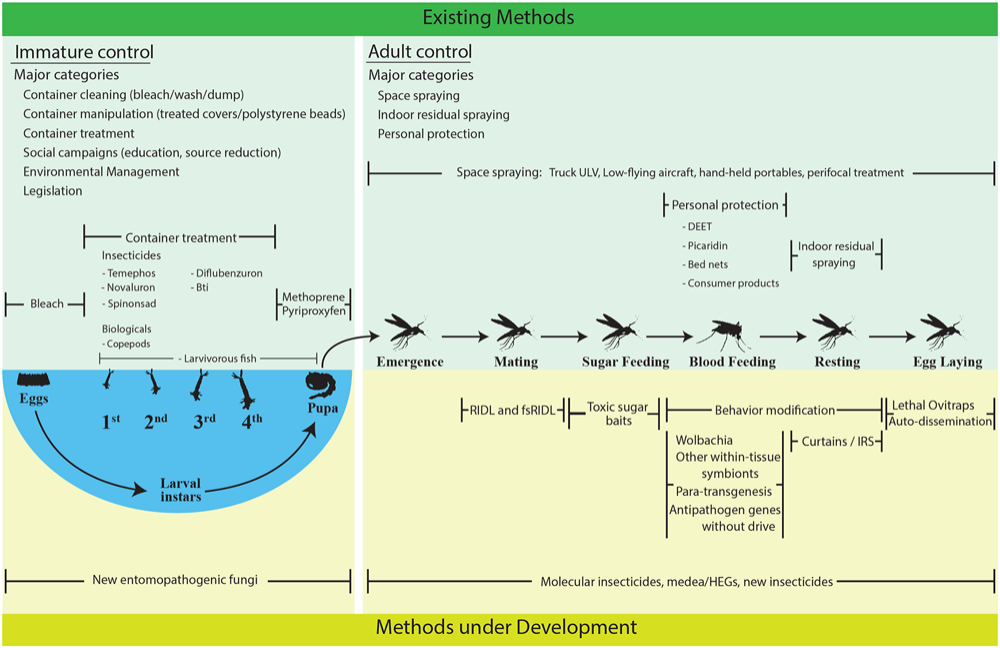
Existing and developing control methods. Existing methods (upper green region) and methods under development (lower yellow region) are enumerated and separated by those that affect larval mosquito stages (left) and those that affect adult mosquito stages (right). Methods that target a particular sub-stage within a mosquito’s life cycle are oriented vertically with those sub-stages.

Although the concept of integrated intervention for dengue prevention is gaining increasingly broader acceptance, to date, no consensus has been reached regarding the details of how and what combination of approaches can be most effectively implemented to manage disease. To fill that gap, the PDC proposed a three step process: (1) a critical assessment of current tools and those under development, (2) outlining a research agenda for determining, in a definitive way, what existing tools work best, and (3) determining how to combine the best vector control options, which have systematically been defined in this process, with DENV vaccines. The PDC convened a meeting of international experts during November 2013 in Washington, DC, to address the first step, i.e., critically review existing interventions and tools under development (Box 2 and Fig 1).

**Box 2. Key Papers from Vector Control for Dengue Prevention**

- Egger JR, Ooi EE, Kelly DW, Woolhouse ME, Davies CR, Coleman PG. Reconstructing historical changes in the force of infection of dengue fever in Singapore: Implications for surveillance and control. Bulletin of the World Health Organization. 2008; 86(3): 187–196. doi:10.2471/blt.07.040170.
- Erlanger TE, Keiser J, Utzinger J. Effect of dengue vector control interventions on entomological parameters in developing countries: A systematic review and meta-analysis. Med Vet Entomol. 2008; 22(3): 203–221. doi:10.1111/j.1365-2915.2008.00740.x.
- Scott TW, Morrison AC. Vector dynamics and transmission of dengue virus: Implications for dengue surveillance and prevention strategies: vector dynamics and dengue prevention. In: Rothman AL, editor. Dengue virus. Berlin: Springer-Verlag Berlin; 2010.pp. 115–128. doi:10.1007/978-3-642-02215-9_9.
- Gubler D. Dengue, urbanization and globalization: The unholy trinity of the 21st century. Int J Infect Dis. 2012; 16E2-E2. doi:10.1016/j.ijid.2012.05.009.
- World Health Organization. Sustaining the drive to overcome the global impact of neglected tropical diseases: Second WHO report on neglected diseases. Geneva: World Health Organization; 2013.

Deliberations fell into three broad categories: (1) current state-of-the-art (existing) interventions, (2) new tools and strategies (methods under development), and (3) mosquito ecology and modeling. For existing interventions, assessment included which life stage was targeted and evidence establishing the degree to which the intervention impacts mosquito populations and DENV transmission (S1 Table). Expectations of impact were based on available data from small-scale experiments, large-scale field trials, and model results. For interventions under development, assessment included progress within a product developmental pipeline (S2 Table). Best circumstances were outlined for successful deployment of all interventions and challenges faced in contexts where an intervention has already been introduced or is being considered was outlined (S3 Table). Although rigorous economic assessment of each intervention was outside the scope of this workshop, general issues related to ways that development, delivery, and cost could be improved for each intervention were explored (S4 Table). Cost-effectiveness will be fundamentally important in the future for selection and implementation of any vector control intervention.

### Existing Tools and Strategies

A list of currently recommended vector control interventions was generated from WHO guidelines for dengue control (S1 Table) [4]. Topical repellents and legislation (regulation) were added to the list. To streamline discussion, interventions were categorized by tools that: (1) offer best sustained control, (2) offer best epidemic mitigation, or (3) are politically driven, but may not provide substantial benefit to reduced disease. The primary assessment criterion of a single existing strategy was expert opinion regarding evidence of adult vector population reduction. The discipline of vector control has been strongly influenced by the theory developed by Ronald Ross and George Macdonald (i.e., the Ross-Macdonald model), which asserts that the potential for mosquito-borne pathogen transmission is largely dependent on adult vector mosquito abundance, survival through the pathogen incubation period, and human-biting rate [36]. Interventions that reduce adult mosquito population density, daily probability of survival, and mosquito contact with humans are, therefore, expected to have the biggest impact on decreasing virus transmission. It should be noted, however, that the Ross-Macdonald model was not formulated to specifically explore larval mosquito control. Recent quantitative assessments indicate that under certain circumstances larval control may result in larger than previously expected reductions in pathogen transmission [37].

### Sustained Management

Tools and strategies recommended by panel participants for sustained dengue mitigation included indoor spraying (preferably with residual insecticides) and perifocal spraying with residual insecticides to kill adult mosquitoes [38]. This is supported by results from the perifocal residual insecticide treatment of receptacles for the control of larvae and resting adult mosquitoes in two successful *Ae*. *aegypti* eradication programs in the Northern Territory of Australia from 2004 to 2006 (Tennant Creek) and from 2006 to 2008 (Groote Eylandt) [39]. Perifocal spraying, complimented with source reduction, was the mainstay of the eradication campaign in the Americas. In the Cayman Islands, perifocal spraying was successfully combined with larviciding, as well as intra- and extra-domiciliary spraying [40]. Although perifocal spraying of containers that are potential larval development sites with residual insecticide is frequently promoted in literature on dengue prevention [41], indoor residual insecticide application has only been carried out on a few occasions as part of a top-down approach for dengue vector control [42]. Studies of indoor residual spraying (IRS) in Cairns, however, indicate that the strategy, when used appropriately, (1) reduces adult female density [43] and (2) significantly reduces DENV transmission risk [44].

Comprehensive container larvicide treatment (with insecticides or biologicals) and container removal are recommended for sustained management of immatures, and, thus, indirectly adult *Aedes*, based on demonstration of small-scale successes using combinations of interventions [45]. Social mobilization campaigns (education, public relations) [46,47], environmental management [48], and legislation (enforcement and incentives) were considered effective as components of sustained mitigation programs [14]. Failure of dengue vector control strategies has often been associated with the absence of active local community involvement [49].

Conversely, two highly visible interventions (aerial and truck mounted ultra-low volume [ULV] space-spraying) were not recommended due to lack of impact on mosquito population reduction and lack of cost-effectiveness for routine delivery [50].

Improving sustained mitigation will require a “career structure” that maintains trained staff through inter-epidemic and epidemic periods. Lack of personnel with appropriate technical expertise plagues many dengue prevention programs [51]. This could be addressed by generating permanent positions that, through financial or promotional gains, ensure continuity in operational implementation. Establishing staff work schedules that fit community lifestyles would facilitate visits to homes and other structures when residents/owners are most likely to be present and allow access.

### Epidemic Mitigation

Epidemic response tools and strategies can reduce dengue cases. When applied early in the transmission season (i.e., during the increase of epidemic cases), indoor ULV insecticide applications were associated with a reduction in the number of dengue cases in Iquitos, Peru [20]. Similarly, dengue epidemics in Brazil and Hawaii were controlled using indoor spraying with residual insecticide [52]. The challenge for this approach is early recognition of increased risk followed quickly by comprehensive broad scale intra-domicile insecticide application. This is difficult, if not impossible, to accomplish in complex mega-cities. Improved surveillance efforts that guide more logistically feasible spatial and temporal implementation of vector control are needed to improve the speed of response and to elevate the public health impact of targeted epidemic suppression strategies [19]. In most circumstances, as for sustained management, spraying with low-flying aircraft was not considered a viable approach for epidemic mitigation of dengue.

Under certain specific circumstances, in conjunction with indoor residual spray, personal repellents (e.g., DEET) and insecticide treated material inside homes (e.g., bed nets and/or treated lining) where *Ae*. *aegypti* rest and bite humans were recognized as a potential component of epidemic mitigation. Laboratory studies have shown that *Ae*. *aegypti* and *Ae*. *albopictus* infected with the four DENV serotypes respond similarly to uninfected mosquitoes indicating that repellents containing DEET may be effective against infective mosquitoes in the field [53].

Field studies in Mexico demonstrated reduction in vector populations using pyrethroid treated curtains and water jar covers [54]. Data, however, are lacking to assess to what extent these interventions reduce dengue cases during an epidemic.

At the local level, emergency legislation for immediate access to vacant lots, households, schools, and/or offices may be required, where not currently enacted, to allow comprehensive attention to key virus transmission sites.

Perhaps the most important point derived from panel discussions is that the primary reason for the inability to recommend a specific existing intervention for either sustained management or epidemic mitigation is the absence of data providing clear evidence of a direct, positive health impact, i.e., reduced human dengue cases. For example, no panelist was aware of any controlled study carried out to evaluate the protective efficacy of ULV spraying against human DENV infection despite the fact that ULV spraying continues to be a frequently used intervention for dengue. The group recognized that selection and impact of existing, or any, vector control strategy to mitigate vector populations and/or the overall impact on dengue will be site-specific based on underlying human, virus, and/or vector dynamics, which is consistent with the requirement for an integrated approach [54]. Political pressures, however, typically drive implementation of highly visible activities, such as aerial, truck-mounted, and space-spraying, without a solid empirical basis for using these approaches to reduce human infection and/or disease.

### Interventions Currently under Development

Because, as currently used, existing tools for suppressing dengue vector populations are often judged ineffective, there has been increasing interest in developing new tools. Some are being developed by technological improvement of existing approaches. Others involve conceptually novel approaches that have emerged from breakthroughs in biotechnologies.

Meeting participants developed a list of tools and strategies in development for dengue suppression (S2 Table) that are designed to (1) reduce the overall mosquito population, (2) change the age structure of the female mosquito population, (3) manipulate female mosquito behavior, (4) replace wild-type mosquitoes with strains/genotypes that do not transmit DENV, or (5) some combination of the above. Each tool was evaluated based on criteria related to the (1) current stage of development, (2) predicted efficacy, (3) expected limitations, and (4) potential for integration with other tools.

### Population Reduction

Synthetic insecticides have long been used to suppress mosquito populations, but negative environmental and health impacts, lack of intrinsic efficacy, and evolution of insecticide-resistant mosquitoes are challenging current insecticide-reliant strategies [55–59]. Two major paths for alleviating these problems come from investment in new chemical classes of insecticides [60] and “molecular insecticides” that use specially engineered nanoparticles to target insecticides at specific insect tissues and protect the active ingredient from environmental degradation [61]. There have been major investments in developing and testing new insecticides within existing chemical classes (and in new classes) with low off-target effects and long residual efficacy. Some of the first products from these efforts are expected to be field tested in the near future, and completely novel compounds should soon follow. The goal is to have a tool(s) that is as effective at suppressing DENV transmission as DDT was (high kill and/or repellent with effectiveness for six months or more), but without associated health or environmental impacts. Early indications are encouraging, but it is too soon to draw firm conclusions about the potential for reaching that goal. As with any intervention that poses a potential selection pressure on mosquito populations, behavioral or physiological, monitoring for change in efficacy over time will be a vital part of the strategy.

There are a number of tools in the pipeline that involve using insect genetic engineering for population reduction. The one furthest in development is an *Ae*. *aegypti* strain engineered by the start-up company, Oxitec, that has conditional lethality (RIDL^R^) [62]. The genetically engineered strain can be reared to high numbers in captivity simply by adding tetracycline to the larval diet. When transgenic males are released into wild-type mosquito populations in the field and mate with normal females, almost 100% of the offspring are expected to die during late larval development stages. Data from small-scale trials indicate that wild populations can be suppressed, but suppression takes a number of months and requires continual release of the transgenic strain. This approach is expected to work best when the initial population is at low density and the treatment area is relatively small. Thus, this approach could be used with other tools that decrease vector population size before RIDL^R^ mosquitoes are released. Promising results were obtained from field trials in Malaysia and the Cayman Islands [63,64] and small Brazilian communities [65,66]. As for any transgenic mosquito strain, public acceptance and regulatory approval will be required.

A public/private partnership (Gates/Oxitec) developed a different transgenic *Ae*. *aegypti* strain (OX36404C) in which only the females die (fsRIDL) when tetracycline is removed from their larval diet [67]. Such a strain could be more effective than one in which both sexes die. Although indoor large-cage trials were successful [68], results from an outdoor field-cage trial in Mexico were disappointing [69]. Lowered mating competitiveness of transgenic males was one cause of the failure of that trial. It is possible that new strains with this female-killing transgenic construct may have better performance. Detailed population dynamics models indicate that mosquito suppression with this female-killing technique may be problematic in heterogeneous city settings and that attention must be paid to developing effective male release methods [70]. Some researchers also question the feasibility of RIDL for large cities or large geographic areas due to logistic challenges of delivery and concerns about cost. The developers, however, argue that these obstacles can be overcome [71].

A conceptually similar, but methodologically different, biotechnology approach for population reduction would entail multiple releases of male *Ae*. *aegypti* artificially infected with a strain of a microbe, *Wolbachia*, that renders them 100% reproductively incompatible with wild-type females [72]. If incompatibility was less than 100%, releases could presumably result in resurgence of a mosquito population carrying the *Wolbachia* (see below).

Another transgenic method based on sex ratio distortion exploits the specificity of the homing endonuclease I-Ppol, and is in early stages of development in *Anopheles* mosquitoes [73]. Using this approach, a mosquito strain was developed that produces >95% male offspring and has been shown to suppress caged wild-type mosquitoes following multiple releases. This approach of sex ratio distortion may provide the foundation for self-perpetuating control of *Anopheles* if the DNA sequence causing sex distortion can be inserted on the Y-chromosome. In the future, it may be possible to develop similar methods for *Ae*. *aegypti* that could suppress wild *Ae*. *aegypti* populations based on a single release of a transgenic strain. This is a more daunting challenge with *Ae*. *aegypti* because it lacks a distinct sex chromosome.

In the past few years, a new tool for insect transgenesis and genome editing has emerged. This tool, based on CRISPR/Cas9 DNA double-strand breaks and repair, has the potential to substantially propel the field of genetic pest management forward [74,75]. Unlike homing endonucleases, in which changing the DNA target site requires major protein engineering, the CRISPR/Cas9 system enables targeting based simply on changing the nucleotide sequences coding for guide RNAs. This transforms the whole process of gene manipulation into one that is much more efficient and less costly. The CRISPR/Cas9 approach could be used for building self-perpetuating, sex-biasing *Aedes* strains or strains that drive a genetic load into an *Aedes* population strong enough to cause substantial decline in density, or local elimination.

Attracting and then killing female mosquitoes as they lay eggs, via lethal ovitraps or sticky gravid traps, is a targeted, alterative strategy for vector population reduction first recognized in the 1970s [76]. Early attempts in Brazil [77] and Thailand [78] demonstrated reduction in *Ae*. *aegypti* adult and larval populations, but did not meet expectations required for elimination. More efficient trap designs are currently being developed and evaluated [79]. This includes a new lethal ovitrap design that was coupled with a bacteria formulation that produces volatile compounds that are attractive to ovipositing female *Ae*. *aegypti* [80–82]. A recent field trial in Peru generated encouraging preliminary results [83]. Lethal killing stations utilizing adhesives have also been shown to reduce gravid *Ae*. *aegypti* by ~80% in Puerto Rico [79]. Larger, more comprehensive trials with epidemiologic outcomes are planned.

Other population reduction approaches in early development include mosquitocidal fungus [84,85]. Mathematical models are being used to assist in optimizing vector population reduction tools and strategies [86,87]. Early tests of curtains impregnated with insecticide to reduce mosquito populations in Guatemala were encouraging [54], but a subsequent trial in Thailand was not successful [88]. Differences in results may have been due to differences in the structure of houses at the two study locations.

### Altered Age Distribution

One strategy, involving insertion of the wMelPop strain of *Wolbachia* into *Ae*. *aegypti*, aims at shifting the vector population age-structure by reducing the number of females that live long enough to become infectious and transmit DENV [89]. Output from mathematical models indicates that such a *Wolbachia* strain could reduce dengue transmission [90]. Field tests in Australia and Vietnam were, however, disappointing because high fitness costs of the wMelPop strain to infected *Ae*. *aegypti* prevented its establishment [91,92]. New life-shorting strains are in development, but are not as encouraging as *Wolbachia*-transfected virus refractory strains discussed below. Transgenic gene-drive constructs tightly linked with effector genes that shorten life span have been discussed as a control strategy, but none are currently in development.

### Behavior Modification

The use of behavior-modifying chemicals and products, such as spatial repellents, that deter blood-seeking mosquitoes from humans, and, thus, reduce or prevent pathogen transmission, is receiving increased research attention [93]. Unlike the traditional use of chemicals that are designed to kill following contact of vectors with a treated surface, these active ingredients are volatile and are released into a space, such as inside a home, to prevent mosquito entry and/or disrupt sensory perception required to detect and locate a human host [93,94]. These same products may also interfere with indoor resting behavior, thereby further enhancing the effect on adult vector populations due to mosquitoes being forced to search and rest under suboptimal environmental conditions required for survival. In Australian houses, metofluthrin has been shown to rapidly reduce biting and, in some cases, kill adult *Ae*. *aegypti* without expelling them through open windows [95,96]. Field studies in Vietnam have shown densities of adult *Ae*. *aegypti* inside homes treated with metofluthrin plastic latticework strips to be significantly lower than those in untreated houses for six weeks [97]. Randomized, cluster-controlled trials using mosquito coils containing either transfluthrin or metofluthrin in China and Indonesia, respectively, have demonstrated protective efficacy against new malaria infections with an associated reduction in vector densities and biting rates at sentinel homes, respectively [98,99]. A similar, small-scale field trial combining both entomological and epidemiological metrics will begin soon to evaluate the impact of a similar spatial repellent product to interfere with mosquito-human contact in a DENV transmission setting.

### Refractory Strains/Genotypes

The most promising near-term approach for rendering mosquito populations incapable of DENV transmission involves artificial infection with a strain of *Wolbachia* that does not significantly impact mosquito life span and, thus, has high fitness, allowing for rapid field establishment [69]. Field trials in Australia and Vietnam are underway, and the strain is well established in several Australian locations [91,100–106]. Potential for the spread of this strain is being evaluated and new strains are in development [91]. Field trials to assess the epidemiologic impact of *Wolbachia*-transfected *Ae*. *aegypti* on DENV transmission began recently.

An alternative approach is engineering a DNA sequence into the mosquito’s genome that, when transcribed, produces an RNA molecule that blocks DENV replication. An RNAi transmission-blocking strain for DENV-2 was developed and evaluated in the laboratory [107,108]. No field tests have been conducted. Mathematical models indicate that spreading this transgene into wild populations would require far fewer lab-reared mosquitoes than the conditional lethal RIDL^R^ approaches, but would likely require continuous releases lasting about one year [109,110]. These would need to be followed by intermittent releases if there was a small fitness cost associated with the gene for refractoriness [111]. The current strain only inhibits one of the four DENV serotypes. Efforts are underway to develop a transgenic strain that does not transmit any of the DENV serotypes.

Instead of spreading anti-dengue genes into populations through repeated releases, transgenic approaches could potentially link an anti-dengue gene with a gene-drive mechanism that pushes the anti-dengue gene into the mosquito population based on super-Mendelian inheritance. One long-term effort involves a gene drive mechanism, Medea, in which all offspring from a female die if they do not inherit the gene-drive sequence and the linked anti-dengue gene [112]. Although this and other gene-drive mechanisms have worked in fruit flies, to date, they have not been successful in *Ae*. *aegypti*. The recent emergence of the CRISPR/Cas9 methodology [74,75], discussed above, has the potential to reinvigorate research aimed at driving anti-dengue genes into *Aedes* populations.

### Considerations regarding Entomological Endpoints of Health Impact

Panel members recognized that critical to the evaluation of new vector control strategies currently under development, as well as a limitation of previous efficacy trials, has been the methodological challenges of documenting changes in vector densities or other entomological endpoints. Surveillance for *Aedes* vectors of dengue has historically concentrated on immature stages, such as larvae and pupae [113]. This has ranged from container-based indices, such as the Breteau Index and House index, to pupal demographic surveys. With the exception of pupal surveys, immature container indices have generally failed to correlate well with adult populations, let yet alone dengue risk [114–116]. Often container and pupal surveys are compromised by unknown cryptic or inaccessible containers, such as subterranean pits/tanks [117,118] and roof gutters [119], that can serve as major *Aedes* producers. Recent emphasis has shifted to the measure of adult populations, especially female *Ae*. *aegypti*. The advent of adult sampling methods such as aspirators [120,121], Biogents Sentinel traps [122], and gravid traps [123–125] allow for relatively simple and inexpensive means to measure actual adult populations. Measurement of adult female populations provides a much more direct assessment of the impact of interventions on risk of human DENV infection than do immature mosquito surveys [113,114]. Furthermore, adult females that are captured can be assayed for virus and biocontrol agents, such as *Wolbachia* [125]. The highly variable and clumped distribution of *Ae*. *aegypti* indicates, however, that trials need to be sufficiently powered with stratified designs to capture account for this variability. When considering design, the techniques described above fall into two major categories: house-to-house surveys and fixed trap methods. Survey methods, although more susceptible to operator variation, can often cover more houses per unit time (often better spatial coverage) than fixed trap methods, which better capture short-term temporal variation.

### Summary and Future Directives

Vector control for dengue can be effective, but, in order for that to happen, implementation must be done thoroughly, comprehensively, and be sustained. Deliberations at this workshop were the first in a three-step process that will develop a framework to guide decision-making on the effective application and integration of dengue vector control interventions. Updating the assessment of vector control options is desirable given the breadth of currently available vector control tools; the increasing number of new interventions under development; expectations that a dengue vaccine will be commercially available in the near future; and the seemingly unabated, expanding burden of dengue illness.

Decreasing dengue will require increased capacity for informed preventive, versus the current reliance on reactive, vector control efforts that use existing and/or novel interventions with improved, more efficient delivery systems. It is time to stop using interventions simply because they have been used in the past or because they are politically popular. Many of the current *Ae*. *aegypti* control methods continue to be deployed simply for community visibility without evidence that they prevent disease. The public health cost—benefit of existing tools and strategies needs to be more clearly defined to reduce dengue at regional and global scales. Limited resources in endemic countries must be more efficiently targeted to only those essential activities that will have an epidemiologic impact on specific virus transmission scenarios, even if this requires policy changes and results in implementation challenges. For example, given that the vector control strategies for dengue and chikungunya viruses are broadly similar, the recent and rapid geographic expansion and intensified transmission of chikungunya virus has cost—benefit implications for integration of vector and disease management. In many areas, it may be possible to simultaneously tackle both diseases with the same or similar tools and strategies.

Many of the *Ae*. *aegypti* control strategies in development will have time-lagged impacts on adult populations, i.e., *Wolbachia* and transgenics. Those kinds of interventions will be best applied in sustained, proactive implementation and will likely be inadequate for rapid control of a developing epidemic. In addition to such proactive strategies, dengue prevention will benefit from enhanced capacity for outbreak response, before epidemics have peaked and begun to decline on their own. In this regard, spatial repellents, molecular insecticides, and indoor insecticide application may be particularly amenable to swift deployment with high public health impact.

To reach their full potential for all interventions, proactive and reactive, will require additional thinking about the conceptual basis for strategic innovation, updated guidelines for rigorous assessment of intervention options, and an expanded prevention toolbox that is supported by solid empirical data. Funding agencies that are requiring systematic testing and assessment of novel interventions through the product development pipeline are facilitating these changes. Conversely, most existing interventions were developed in an era when epidemiologic outcomes were not emphasized. Understanding when and how existing tools (i.e., indoor application of existing insecticides) can be best applied will require a shift in attention toward these issues and, interestingly, can benefit from lessons learned from the newer interventions currently in development.

Predicting the effectiveness of existing tools is not currently possible because almost nothing is known about how well they prevent disease. There is a vital need for robust, rigorously designed field trials with epidemiological and entomological outcomes to improve understanding of how well existing tools can reduce DENV transmission and their optimal implementation. Only then can strategy-specific programs be realistically developed and evaluated. Mathematical models indicate that spatially or temporally targeting existing vector control interventions may be more effective than applying them evenly across different locations and times [86,126,127]. Likewise, targeting control on mosquito life stages other than adults (larvae and pupae) may be more effective than previously thought based on the Ross-Macdonald theory [37]. These theoretical advances are helpful, but they are not a replacement for results from field studies and, therefore, new theories require empirical validation.

Among the greatest challenges in dengue vector control is scaling-up local, small-scale successes. Ideally, the aim will be to implement an effective intervention, or combination of interventions, across modern mega-cities as well as large geographic areas that encompass entire countries and transcend national boundaries. The current inability to effectively scale-up and predict the public health impact over large geographic areas explains much of the failure of current dengue prevention programs, despite their potential to be effective at small scales. This roadblock must be overcome. Unless effective control can be scaled-up for mega-cities and regional control, the dengue burden will continue to grow.

There is a growing consensus among dengue experts that eliminating dengue as a public health burden can only be achieved by integrating vector control with vaccines. Integrated strategies will pose new challenges, such as (1) designing vector control strategies that account for variation in spatial and temporal patterns in vaccine rollout and the subsequent alterations that the vaccine may cause in virus transmission dynamics, (2) effective integration of entomologic measures in vaccine trials to guide site characterization and measures of trial impact on disease, (3) integrating epidemiological indexes to measure the capacity of vector control interventions to prevent disease, and (4) designing surveillance systems that can distinguish the epidemiologic impact of vaccines versus vector control. Addressing these issues will be a central part of developing a research agenda for assessing the epidemiological impact of the top vector control candidates, alone and in combination with a DENV vaccine.

## Supporting Information

S1 Table Evidence of impact of existing interventions on *Aedes aegypti* and dengue virus. Abbreviations: *Dengue: guidelines for diagnosis, treatment, prevention and control, World Health Organization 2009; ** RCT, randomized cluster trial; SS, small scale field evaluations (<1,000 houses in both intervention and control arms; short duration); LS, large scale field evaluation (>1,000 houses in both intervention and control arms; long duration); RE, relative effectiveness (from Erlanger et al. 2008) 1-relative reduction of density index (BI, CI, HI in all cases cited), 0 indicates elimination whereas 1 indicates no difference between intervention and control; ***unpublished data.
(DOCX)

S2 Table Stage of development of developing interventions. Green cells indicate that an assessment has been completed successfully, whereas yellow cells indicate that an assessment is currently in progress.
(DOCX)

S3 Table Best circumstances for successful deployment of interventions and challenges faced in contexts where the intervention has already been introduced or is being considered. Abbreviations: E, existing intervention; D, intervention under development; NA, not applicable as intervention testing is currently underway or has yet to be tested under controlled field study designs and/or implemented in public health program.
(DOCX)

S4 Table Ways that development, delivery, and cost could be improved for each intervention. Abbreviations: E, existing intervention; D, intervention under development.
(DOCX)
