# Supplementary material for: A Critical Assessment of Vector Control for Dengue Prevention
Source: PLoS Negl Trop Dis. 2015 May 7;9(5):e0003655. doi: 10.1371/journal.pntd.0003655 (PMC4423954; doi:10.1371/journal.pntd.0003655)
Supplement: S1 Table — Abbreviations: *Dengue: guidelines for diagnosis, treatment, prevention and control, World Health Organization 2009; ** RCT, randomized cluster trial; SS, small scale field evaluations (<1,000 houses in both intervention and control arms; short duration); LS, large scale field evaluation (>1,000 houses in both intervention and control arms; long duration); RE, relative effectiveness (from Erlanger et al. 2008) 1-relative reduction of density index (BI, CI, HI in all cases cited), 0 indicates elimination whereas 1 indicates no difference between intervention and control; ***unpublished data. (DOCX) [file pntd.0003655.s001.docx]

| **Intervention*** | ***Aedes aegypti*** | | |  | **Dengue virus** | | |
| --- | --- | --- | --- | --- | --- | --- | --- |
|  | **Evidence of impact^**^** | **Key studies or sites** | **Mode of ___action** |  | **Evidence of impact** | **Key studies or sites** | **Mode of action** |
| **Larval control** |  |  |  |  |  |  |  |
| *Container manipulation (polystyrene beads, lids)* | Yes, not controlled | - | Prevent oviposition |  | No | - | Reduced density |
| *Container treatment (chemical)* | |  |  |  |  |  |  |
| a. Temephos | Yes  SS, LS  RE=0-0.24 | India[^2^](#_ENREF_2)  Thailand[^3^](#_ENREF_3) | Killing |  | Yes, not controlled  RE = 0.47 | Cambodia[^4^](#_ENREF_4) | Same |
| *Container treatment (non-chemical)* | |  |  |  |  |  |  |
| a. Diflubenzuron | Yes, not controlled | - | Killing |  | Same | - | Same |
| b. Methoprene | Yes, SS | Indonesia[^5^](#_ENREF_5) | Same |  | Same | - | Same |
| c. Novaluron | Same | - | Same |  | Same | - | Same |
| d. Spinosad | Same | - | Same |  | Same | - | Same |
| e. Bti | Same | - | Same |  | Same | - | Same |
| f. PPF | Yes,  SS | Brazil** | Same + sterilization |  | - | - | Same |
| *Container treatment (biologicals)* | |  |  |  |  |  |  |
| a. Larvivorous fish | Yes, SS  RE=0-0.40 | Mexico[^6^](#_ENREF_6)  Thailand[^7^](#_ENREF_7)  China[^8^](#_ENREF_8) | Killing |  | No | - | Same |
| b. Copepods | Same  RE=0 | Vietnam[^9^](#_ENREF_9) | Same |  | Same | - | Same |
| c. Dragonfly larvae (*Crocothermeis servilia)* | Same  RE=0.05 | Myanmar[^10^](#_ENREF_10) | Same |  | Same | - | Same |
| *Community-based* |  |  |  |  |  |  |  |
| a. Education campaigns | Yes | Puerto Rico[^11^](#_ENREF_11) | - |  | Yes, controlled | - | Same |
| b. Source reduction campaigns | Yes | Honduras[^12^](#_ENREF_12) | - |  | - | - | Same |
| c. Social mobilization | Yes  RCT | Nicaragua/ Mexico^**^ | Container mgmt. |  | Yes  RCT | Nicaragua/ Mexico* | Same |
| *Environmental management* | |  |  |  |  |  |  |
| a. Manipulation (waste removal) | Yes, not controlled | - | Breeding site removal |  | Yes, not controlled | - | Same |
| **Adult control** |  |  |  |  |  |  |  |
| *Space spraying* |  |  |  |  |  |  |  |
| a. Truck ULV | Yes, not controlled | - | Killing |  | Yes, not controlled | - | Reduced density |
| b. Low-flying aircraft | Same | - | Same |  | Same | - | Same |
| c. Hand-held / backpack portables indoors | Same | - | Same |  | Same | - | Same |
| d. Perifocal treatment with residuals | Same | - | Same |  | Same | - | Same |
| *Indoor residual spray* | Same | Australia^13,14^ | Same |  | Same | - | Same |
| *Lethal ovitraps* | Same | Thailand, Australia^15^  Brazil | - |  | - | - | - |
| *Personal protection* |  |  |  |  |  |  |  |
| a. DEET | No | - | Repel |  | No | - | Reduced biting |
| b. Picaridin | Same | - | Same |  | Same | - | Same |
| c. Bed nets | Same | - | Killing |  | Same | - | Reduced density |
| d. Consumer products | Yes, not controlled | Several | Deterrence, killing |  | Same | - | Reduced biting |

1. Erlanger TE, Keiser J, Utzinger J. Effect of dengue vector control interventions on entomological parameters in developing countries: a systematic review and meta-analysis. Med Vet Entomol 2008;22:203-21.

2. Geevarghese G, Dhanda V, Ranga Rao PN, Deobhankar RB. Field trials for the control of *Aedes aegypti* with Abate in Poona city and suburbs. Indian J Med Res 1977;65:466-73.

3. Bang YH, Pant CP. A field trial of Abate larvicide for the control of Aedes aegypti in Bangkok, Thailand. Bull World Health Organ 1972;46:416-25.

4. Suaya JA, Shepard DS, Chang MS, et al. Cost-effectiveness of annual targeted larviciding campaigns in Cambodia against the dengue vector Aedes aegypti. Tropical medicine & international health : TM & IH 2007;12:1026-36.

5. Houten AT, Aminah NS, Gratz NG, Mathis HL. Pilot Trial with Methopren (OMS 1697) against *Aedes aegypti* in Jakarta, Indonesia. Geneva, Switzerland: World Health Organization; 1980.

6. Martínez-Ibarra JA, Guillén YG, Arredondo-Jiménez JI, Rodríguez-López MH. Idigenous fish species for the control of *Aedes aegypti* in water storage tanks in Southern México. BioControl 2002;47:481-6.

7. Phuanukoonnon S, Mueller I, Bryan JH. Effectiveness of dengue control practices in household water containers in Northeast Thailand. Tropical medicine & international health : TM & IH 2005;10:755-63.

8. Wu N, Wang SS, Han GX, Xu RM, Tang GK, Qian C. Control of Aedes aegypti larvae in household water containers by Chinese cat fish. Bull World Health Organ 1987;65:503-6.

9. Kay BH, Nam VS, Tien TV, et al. Control of aedes vectors of dengue in three provinces of Vietnam by use of Mesocyclops (Copepoda) and community-based methods validated by entomologic, clinical, and serological surveillance. The American journal of tropical medicine and hygiene 2002;66:40-8.

10. Sebastian A, Sein MM, Thu MM. Supression of *Aedes-aegypti* (DIPTERA, CULICIDAE) using augmentative release of dragonfly larvae (ODONATA, LIBELLULIDAE) with community participation in Yangon, Myanmar. BULLETIN OF ENTOMOLOGICAL RESEARCH 1990;80:223-32.

11. Winch PJ, Leontsini E, Rigau-Perez JG, Ruiz-Perez M, Clark GG, Gubler DJ. Community-based dengue prevention programs in Puerto Rico: impact on knowledge, behavior, and residential mosquito infestation. The American journal of tropical medicine and hygiene 2002;67:363-70.

12. Leontsini E, Gil E, Kendall C, Clark GG. Effect of a community-based Aedes aegypti control programme on mosquito larval production sites in El Progreso, Honduras. Trans R Soc Trop Med Hyg 1993;87:267-71.

13. Vazquez-Prokopec GM, Kitron U, Montgomery B, Horne P, Ritchie SA. 2010. Quantifying the spatial dimension of dengue virus epidemic spread within a tropical urban environment. PLoS Negl Trop Dis 4(12): e920. doi:10.1371/journal.pntd.0000920

14. Ritchie SA, Hanna JN, Hills SL, Piispanen JP, McBride WJH, et al. 2002. Dengue control in north Queensland, Australia: case recognition and selective indoor residual spraying. Dengue Bulletin 26: 7–13.

15. Ritchie SA, Long S, Smith G, Pyke A, and Knox T. 2004. Entomological investigations in a focus of dengue transmission in Cairns, Queensland, Australia using the sticky ovitrap. J. Med. Entomol. 41: 1-4.
