# Supplementary material for: A Critical Assessment of Vector Control for Dengue Prevention
Source: PLoS Negl Trop Dis. 2015 May 7;9(5):e0003655. doi: 10.1371/journal.pntd.0003655 (PMC4423954; doi:10.1371/journal.pntd.0003655)
Supplement: S2 Table — Green cells indicate that an assessment has been completed successfully, whereas yellow cells indicate that an assessment is currently in progress. (DOCX) [file pntd.0003655.s002.docx]

| Intervention | Proof of principle | Lab  expmt | Small-scale field expmt | Small-scale field trial | Large-scale field trial | Target product profile | Impact on DENV infection | Impact on DENV disease |
| --- | --- | --- | --- | --- | --- | --- | --- | --- |
| Larval control |  |  |  |  |  |  |  |  |
| Auto-dissemination |  |  |  |  |  |  |  |  |
| Entomopathogenic fungi |  |  |  |  |  |  |  |  |
| Adult control |  |  |  |  |  |  |  |  |
| Wolbachia (transmission blocking) |  |  |  |  |  |  |  |  |
| Insecticide-treated curtains |  |  |  |  |  |  |  |  |
| Lethal ovitraps |  |  |  |  |  |  |  |  |
| RIDL, fsRIDL |  |  |  |  |  |  |  |  |
| Behavior modification (to include spatial repellents) |  |  |  |  |  |  |  |  |
| Auto-dissemination |  |  |  |  |  |  |  |  |
| Wolbachia (population reduction) |  |  |  |  |  |  |  |  |
| Anti-pathogen genes without drive |  |  |  |  |  |  |  |  |
| Homing endonuclease genes |  |  |  |  |  |  |  |  |
| Reduce and replace |  |  |  |  |  |  |  |  |
| Medea |  |  |  |  |  |  |  |  |
| Underdominance |  |  |  |  |  |  |  |  |
| Molecular insecticides |  |  |  |  |  |  |  |  |
| Within-tissue symbionts (not Wolbachia) |  |  |  |  |  |  |  |  |
