# Supplementary material for: A Critical Assessment of Vector Control for Dengue Prevention
Source: PLoS Negl Trop Dis. 2015 May 7;9(5):e0003655. doi: 10.1371/journal.pntd.0003655 (PMC4423954; doi:10.1371/journal.pntd.0003655)
Supplement: S3 Table — Abbreviations: E, existing intervention; D, intervention under development; NA, not applicable as intervention testing is currently underway or has yet to be tested under controlled field study designs and/or implemented in public health program. (DOCX) [file pntd.0003655.s003.docx]

| **Intervention** | **Stage^1^** | **Best circumstances^2^** | **Challenges where already in use** | **Challenges in new contexts** |
| --- | --- | --- | --- | --- |
| **Larval control** | | | | |
| *Container manipulation (polystyrene beads, lids)* | E | Community awareness & participation in DENV control; knowledge of 'super producer' sites; *Ae*. *aegypti* exclusive vector | Training, maintenance, time of implementation (i.e., surveillance gaps) | Organization |
| *Container treatment (chemical)* | | | | |
| a. Temephos | E | Same | Same | Same |
| *Container treatment (non-chemical)* |  |  |  |  |
| a. Diflubenzuron | E | Same | Same | Same |
| b. Methoprene | E | Same | Same | Same |
| c. Novaluron | E | Same | Same | Same |
| d. Spinosad | E | Same | Same | Same |
| e. Bti | E | Same | Same | Same |
| f. PPF | E | Same | Same | Same |
| g. Auto-dissemination | D | - | - | - |
| *Container treatment (biologicals)* | | | | |
| a. Larvivorous fish | E | Same | Same | Same |
| b. Copepods | E | Same | Same | Same |
| c. Entomopathogenic fungi | D | - | - | - |
| *Community-based* | | | | |
| a. Education campaigns | E | Indicators for people to follow; community awareness & interest; cooperative local government; no tall apartment buildings | Sustainability of interest; time of implementation in relation to epidemics (i.e., surveillance gaps) | Same |
| b. Source reduction campaigns | E | Same | Same | Same |
| *Environmental management* | | | | |
| a. Manipulation (waste removal) | E | Settings with infrastructure to support system | Settings with infrastructure to support system | Same |
| **Adult control** | | | | |
| *Space spraying* | | | | |
| a. Truck ULV | E | Organized campaign; early-warning; starting in high-risk areas | Training, maintenance, time of implementation (i.e., surveillance gaps) | Organization |
| b. Low-flying aircraft | E | Same | Same | Same |
| c. Hand-held / backpack portables indoors | E | Same + indoor biting; ability to apply coverage completely | Same | Same |
| d. Perifocal treatment with residuals | E | Outdoor biting | Same | Same |
| *Indoor residual spray* | E | Indoor biting | Acceptance; SP resistance, laborious | Same |
| *Personal protection* | | | | |
| a. DEET | E | During epidemics; population groups with discretionary spending | User acceptability; willingness to pay (WTP) | Same + Consumer product market |
| b. Picaridin | E | Same | Same | Same |
| c. Bed nets | E | For quarantine purposes; persons who sleep indoors during the day (children); starting in high risk areas; indoor biting; night biting | Same | Same |
| d. Consumer products | E | Population groups with discretionary spending | Same | Same |
| *Wolbachia (transmission blocking)* | D | Dense urban environment; feasible introduction threshold; spatially homogeneous mosquito density; *Ae. aegypti* exclusive vector | Rollout in megacities; large scale production of mosquitoes | Same |
| *Wolbachia (population reduction)* | D | Same | Introduction despite fitness cost | Same |
| *Insecticide-treated curtains* | D | Uniform housing construction | User acceptability; willingness to pay (WTP) | Same; Consumer product market |
| *Lethal ovitraps* | D | Small area; few alternative containers for egg-laying & larval development; a dry season; island, other isolated area; no larvicide resistance | - | - |
| *RIDL, fsRIDL* | D | Dense urban environment; seasonal mosquito density; stable, consistent funding & commitment; island, other isolated area | Community acceptance; regulatory approvals; infrastructure to support rearing and routine releases | Same |
| *Auto-dissemination* | D | - | - | - |
| *Behavioral manipulation (to include spatial repellents)* | D | Enclosed housing structure; strictly indoor biting; no diversion | User acceptability; WTP | Same; Create a consumer-driven product and strategy |
