# Supplementary material for: A Critical Assessment of Vector Control for Dengue Prevention
Source: PLoS Negl Trop Dis. 2015 May 7;9(5):e0003655. doi: 10.1371/journal.pntd.0003655 (PMC4423954; doi:10.1371/journal.pntd.0003655)
Supplement: S4 Table — Abbreviations: E, existing intervention; D, intervention under development. (DOCX) [file pntd.0003655.s004.docx]

| **Intervention** | **Stage** | **Ways to improve development and implementation** | **Ways to improve delivery** | **Factors that would drive cost down** |
| --- | --- | --- | --- | --- |
| **Larval control** |  |  |  |  |
| *Container manipulation (polystyrene beads)* | E | - | Targeted; reduced labor needs | Targeted delivery |
| *Container treatment (chemical)* |  |  |  |  |
| a. Temephos | E | - | Same | Same + cost of active |
| *Container treatment (non-chemical)* |  |  |  |  |
| a. Diflubenzuron | E | - | Same | Same |
| b. Methoprene | E | - | Same | Same |
| c. Novaluron | E | - | Same | Same |
| d. Spinosad | E | - | Same | Same |
| e. Bti | E | - | Same | Same |
| f. PPF | E | - | Same | Same |
| g. Auto-dissemination | D | - | - | - |
| *Container treatment (biologicals)* |  |  |  |  |
| a. Larvivorous fish (*Poecilia reticulata*) | E | - | Same | Same |
| b. Copepods | E | - | Same | Same |
| c. Entomopathogenic fungi | D | - | - | - |
| *Community-based* |  |  |  |  |
| a. Education campaigns | E | Additional evidence of impact; quality control of brigadistas | Target sites of 'super producers' | Community investment; reducing research costs |
| b. Source reduction campaigns | E | - | - | - |
| *Environmental management* |  |  |  |  |
| a. Manipulation (waste removal) | E | - | Where no infrastructure exists | - |
| **Adult control** |  |  |  |  |
| *Space spraying* |  |  |  |  |
| a. Truck ULV | E | - | Targeted; reduced labor needs | Same + maintenance improvement |
| b. Low-flying aircraft | E | - | - | - |
| c. Hand-held / backpack portables indoors | E | - | Same | Same |
| d. Perifocal treatment with residuals | E | - | Same | Same |
| Indoor residual spray | E | Improved residual formulations; non SP formulations | Same | Strategic use to offset laborious application |
| *Personal protection* |  |  |  |  |
| a. DEET | E | - | Integrate with voucher system/subsidized costs | Increased duration; cost of active |
| b. Picaridin | E | - | Same | Same |
| c. Bed nets | E | - | Same | Same |
| d. Consumer products | E | - | User acceptability;  Efficacy against indoor pest mosquitoes: eg., *Culex* | Same + enhanced uptake |
| *Wolbachia (transmission blocking)* | D | Ongoing efforts: non-powered, stackable adult traps and deployment from back of vehicle; cheap, easy diagnostic for Wolbachia infection | - | - |
| *Wolbachia (population reduction)* | D | Same | - | - |
| *Insecticide-treated curtains* | D | New insecticides; evidence of impact; active ingredients; barriers for doors; placement that leverages knowledge of mosquito behavior; synergistic compounds, longer insecticide life; more attractive products for consumers | - | Only 4-8% of cost is in insecticide, so maybe not much scope for lowering cost |
| *Lethal ovitraps* | D | Local production, which is underway and has good prospects wherever there is good support and interest | - | Local production, which is underway and has good prospects wherever there is good support and interest |
| *RIDL, fsRIDL* | D | Improved rearing procedures; better operational support to dynamically identify where to deploy mosquitoes; automated release using GPS | - | Availability of fsRIDL would mean lower cost, more flexible delivery |
| *Auto-dissemination* | D | - | - | - |
| *Behavioral manipulation (to include spatial repellents)* | D | Evidence of impact; combination of top down and bottom up delivery and distribution channels; market surveys for greater end user uptake of products | Integrate with voucher system/subsidized costs | Cost of active; targeted delivery; product duration/replacement scheme |
